# Hederagenin Induces Apoptosis in Cisplatin-Resistant Head and Neck Cancer Cells by Inhibiting the Nrf2-ARE Antioxidant Pathway

**DOI:** 10.1155/2017/5498908

**Published:** 2017-12-31

**Authors:** Eun Hye Kim, Seungho Baek, Daiha Shin, Jaewang Lee, Jong-Lyel Roh

**Affiliations:** ^1^Department of Otolaryngology, Asan Medical Center, University of Ulsan College of Medicine, Seoul, Republic of Korea; ^2^Department of Pathology, College of Korean Medicine, Woosuk University, Jeonju-si, Jeollabuk-do, Republic of Korea

## Abstract

Acquired resistance to cisplatin is the most common reason for the failure of cisplatin chemotherapy. Hederagenin, triterpenoids extracted from ivy leaves, exhibits antitumor activity in various types of cancer. However, the therapeutic potential of hederagenin in head and neck cancer (HNC) has remained unclear. Therefore, we examined the effects of hederagenin in cisplatin-resistant HNC cells and characterized its molecular mechanisms of action in this context. We evaluated the effects of hederagenin treatment on cell viability, apoptosis, reactive oxygen species (ROS) production, glutathione levels, mitochondrial membrane potential (Δ*Ψ*m), and protein and mRNA expression in HNC cells. The antitumor effect of hederagenin in mouse tumor xenograft models was also analyzed. Hederagenin selectively induced cell death in both cisplatin-sensitive and cisplatin-resistant HNC cells by promoting changes in Δ*Ψ*m and inducing apoptosis. Hederagenin inhibited the Nrf2-antioxidant response element (ARE) pathway and activated p53 in HNC cells, thereby enhancing ROS production and promoting glutathione depletion. These effects were reversed by the antioxidant trolox. Hederagenin activated intrinsic apoptotic pathways via cleaved PARP, cleaved caspase-3, and Bax. The selective inhibitory effects of hederagenin were confirmed in cisplatin-resistant HNC xenograft models. These data suggest that hederagenin induces cell death in resistant HNC cells via the Nrf2-ARE antioxidant pathway.

## 1. Introduction

Resistance to chemotherapy is a major obstacle to treating human cancer. Cisplatin is one of the most widely used chemotherapeutic agents in the treatment of various types of solid neoplasms [1]. Cisplatin is currently used as a first-line agent for the treatment of various cancers, including head and neck cancer (HNC), in combination with other anticancer chemotherapeutic agents and/or radiation therapy [1, 2]. However, cisplatin is commonly associated with acquired resistance and increased toxicity, leading to poor tolerance and treatment outcomes [3, 4]. HNC, the eighth most common cancer globally, typically manifests in the oral/nasal cavity, pharynx, and larynx of the upper aerodigestive tract [5, 6]. A combined approach of surgery, radiotherapy, and chemotherapy is commonly used to treat HNC. Nonsurgical chemoradiotherapy has increasingly been used as an organ-preserving treatment for patients with HNC [7, 8]. Recent advances in cancer therapy have improved treatment outcomes; however, survival outcomes in patients with treatment-resistant HNC remain poor. Therefore, improving HNC treatment outcomes requires the development of novel approaches to treat chemotherapy-resistant cancers and identify more effective anticancer agents [7, 9].

Transcription factor nuclear factor (erythroid-derived 2)-like 2 (Nrf2) plays a key role in regulating cellular redox homeostasis because its promoter binds to target genes containing the antioxidant response element (ARE) [10]. Cancer cells buffer cellular reactive oxygen species (ROS) levels by actively upregulating antioxidant pathways, including the Nrf2 pathway, that contribute to cancer therapy resistance [11, 12]. Cellular metabolic pathways and antioxidant defense systems are commonly altered in treatment-resistant cancer cells exposed to high levels of oxidative stress [12, 13]. This metabolic alteration might represent a critical weakness that can be used as a basis to develop therapeutic approaches that selectively kill cancer cells and spare normal cells [14]. Therefore, antioxidant pathways and elevated ROS levels are increasingly gaining acceptance as promising targets in anticancer drug discovery [15].

Hederagenin is a triterpenoid isolated from ivy (*Hedera helix L*.) leaves [16], Chinese sweet tea (*Cyclocarya paliurus*) leaves [17], or other natural products [18]. Accumulating evidence indicates that hederagenin exerts significant cytotoxic effects in several types of cancers. Hederagenin saponin induced apoptosis in various types of human cancer cells by activating components of the mitochondria-mediated intrinsic apoptosis pathway, such as cleaved poly(ADP-ribose) polymerase (PARP), cleaved caspase-3, cleaved caspase-9, and Bax, and by inhibiting the antiapoptotic protein Bcl-2 [18]. In contrast, hederagenin saponin did not significantly affect proteins associated with the extrinsic cell death pathway, such as caspase-8 [16]. Hederagenin has been used as a triterpene template for the discovery of new anticancer compounds [19]. Although these findings suggest that the antitumor activity of hederagenin is mediated by the intrinsic cell death pathway, other mechanisms underlying hederagenin-mediated cancer cell death have yet to be elucidated. The antitumor effects of hederagenin have been examined in the lung, stomach, colon, and breast cancer cells but not in the HNC cells [16–18]. The *in vitro* and *in vivo* efficacy of hederagenin should be more examined in other types of human cancer, particularly those that exhibit resistance to current anticancer treatments. Therefore, we examined the effects of hederagenin in cisplatin-resistant HNC cells and characterized its molecular mechanism of action in this context. We found that hederagenin effectively induced apoptosis in cisplatin-resistant HNC cells *in vitro* and *in vivo* by targeting the Nrf2-ARE antioxidant pathway.

## 2. Materials and Methods

### 2.1. Cell Lines

We evaluated HNC cell lines (AMC-HN2–10), previously established at our institute, as well as SNU-1041, SNU-1066, and SNU-1076 cell lines (Korea Cell Line Bank, Seoul, Republic of Korea). All of the cell lines were authenticated using short tandem repeat-based DNA fingerprinting and multiplex polymerase chain reaction (PCR). The cells were cultured in Eagle's minimum essential medium or Roswell Park Memorial Institute 1640 (Thermo Fisher Scientific, Waltham, MA, USA) supplemented with 10% fetal bovine serum. The cells were maintained at 37°C in a humidified atmosphere with 5% CO_2_. Normal human oral keratinocytes and normal human fibroblasts (HOF) obtained from patients undergoing oral surgery were used for *in vitro* cell viability assays. The cisplatin-resistant HNC cell lines (HN3-cisR, HN4-cisR, and HN9-cisR) were generated by prolonged exposure of the cisplatin-sensitive parental cell lines (HN3, HN4, and HN9 cells, resp.) to increase concentrations of cisplatin (Sigma-Aldrich, St. Louis, MO, USA). The half maximal inhibitory concentration (IC_50_) of cisplatin, as determined using cell viability assays, was 2.2–3.5 *μ*M in the parental HNC cells and 25.5–38.9 *μ*M in the cisplatin-resistant HNC cells.

### 2.2. Cell Viability Assays

Cell viability following exposure to hederagenin (Biobank, Stockport, UK) was assessed using 3-(4,5-dimethylthiazol-2-yl)-2,5-diphenyltetrazolium bromide (MTT) (Sigma-Aldrich), trypan blue exclusion, and clonogenic assays. Control cells were exposed to an equivalent amount of dimethyl sulfoxide (DMSO). Cell viability was also measured in hederagenin- or control-treated cells pretreated with trolox (Enzo Life Sciences Inc., Farmingdale, NY, USA), trigonelline (Sigma-Aldrich), or MG132 (Sigma-Aldrich). MTT assays were performed by incubating the cells with the tetrazolium compound for 4 h followed by solubilization buffer for 2 h. The absorbance at 570 nm was subsequently measured using a SpectraMax M2 microplate reader (Molecular Devices, Sunnyvale, CA, USA). Trypan blue exclusion assays were performed by staining the cells with 0.4% trypan blue and counting the stained cells using a hemocytometer. Clonogenic assays were performed by incubating the cells with 0.5% crystal violet solution and counting the number of colonies (>50 cells) after 14 days of culturing.

Cell death was analyzed by staining the cells with annexin V and propidium iodide (PI) (Sigma-Aldrich). Annexin V- and PI-positive cells were quantitatively analyzed using flow cytometry and Cell Quest Pro software (BD Biosciences, Franklin Lakes, NJ, USA). To measure the mitochondrial membrane potential (Δ*Ψ*m), the cells were stained with 200 nM tetramethylrhodamine ethyl ester (TMRE) (Thermo Fisher Scientific) for 20 min and analyzed using flow cytometry. The median fluorescence intensity (MFI) of each treatment group was normalized to that of the control group. All assays were performed in triplicate using three samples in each assay.

### 2.3. Measuring Glutathione (GSH) Synthesis and ROS Production

Cellular GSH levels were measured in HNC cell lysates using a GSH colorimetric detection kit (BioVision Inc., Milpitas, CA, USA). 2′,7′-Dichlorofluorescein diacetate (DCF-DA) (Enzo Life Sciences, Farmingdale, NY, USA) was used to measure cellular ROS levels in HNC cell lysate supernatants. ROS levels were analyzed using a FACSCalibur flow cytometer equipped with CellQuest Pro software (BD Biosciences).

### 2.4. RNA Interference and Gene Transfection

To silence *SQSTM1*(p62) and *NFE2L2* (Nrf2) expression, cisplatin-resistant HN4-cisR cells were seeded and transfected 24 h later with 10 nmol/L small interfering RNA (siRNA) targeting human *NFE2L2* or *KEAP1* or with a scrambled control siRNA (Integrated DNA Technologies, Coralville, IA, USA). siRNA-induced gene silencing was confirmed using reverse transcription-quantitative PCR (RT-qPCR) analysis of 1-2 *μ*g of total RNA from each sample with a SuperScript® III RT-PCR system (Thermo Fisher Scientific) and Western blot assays with anti-p62 and anti-Nrf2 antibodies. To generate cells stably overexpressing *Nrf2*, HN3 cells were stably transfected with a control plasmid or an *Nrf2*-expressing plasmid (Transomic, Huntsville, AL). *Nrf2* overexpression was confirmed using RT-qPCR and Western blotting.

### 2.5. Western Blot Assays

The cells were plated, grown to 70% confluence, and subsequently treated with the indicated reagents. The cells were lysed at 4°C in radioimmunoprecipitation assay (RIPA) lysis buffer (Thermo Fisher Scientific). A total of 50 *μ*g of protein were resolved using SDS-PAGE on 10%–12% gels, and the separated proteins were transferred to nitrocellulose or polyvinylidene difluoride membranes. The membranes were probed with primary and secondary antibodies. Primary antibodies against the following proteins were used: poly(ADP-ribose) polymerase (PARP) and cleaved PARP, cleaved caspase-3, p53, phospho-p53-Ser15, p62, Bax, Bcl-2, Nrf2, heme oxygenase-1 (HO-1), NAD(P)H: quinone oxidoreductase 1 (NQO1) (Cell Signaling Technology, Danvers, MA), xCT (Abcam, Cambridge, UK), and Keap1 (Santa Cruz Biotechnology, Santa Cruz, CA, USA). *β*-Actin (Sigma-Aldrich) was used as a loading control. All of the antibodies were used at a dilution of 1 : 250–1 : 5000.

### 2.6. Nrf2 Transcriptional Activity

The Nrf2 transcriptional activity was assayed using a Cignal Antioxidant Response Reporter kit (Qiagen, Valencia, CA, USA) according to the manufacturer's instructions.

### 2.7. Immunofluorescence Staining

The cells were incubated with antibodies against p62 and Nrf2. 4′,6-Diamidino-2-phenylindole (DAPI) (Thermo Fisher Scientific) was used as a counterstain to label cell nuclei. The cells were fixed using 3.7% paraformaldehyde in prewarmed complete medium for 15 min at 37°C. The fixed cells were deparaffinized, rehydrated, and incubated with the indicated primary and secondary antibodies. The stained cells were observed and imaged using a fluorescent microscope. Mitochondrial superoxide generation in live hederagenin-treated cells was quantitatively analyzed using mitoSOX (Thermo Fisher Scientific). The stained cells were observed using a fluorescent microscope. The mean fluorescence intensity in each group was normalized to that in the control group.

### 2.8. Tumor Xenografts

All of the animal procedures were performed in accordance with protocols approved by the Institutional Animal Care and Use Committee of our institution. Six-week-old athymic BALB/c male nude mice (nu/nu) were purchased from Central Lab Animal Inc. (Seoul, Republic of Korea). HN9-cisR cells were subcutaneously injected into the flank of nude mice. Beginning on the first day gross nodules from tumor implants were detected, the mice began receiving intraperitoneal injections of the vehicle control or hederagenin (50, 100, or 200 mg/kg daily). Each treatment group included 10 mice. The tumor size and body weight were measured twice a week, and the tumor volume was calculated as length × width^2^/2. The mice were sacrificed on day 35, and the tumors were isolated and analyzed for cellular GSH levels. Apoptosis in tumors was analyzed using an in situ terminal deoxynucleotidyl transferase-mediated dUTP nick-end labeling (TUNEL) assay (Promega, Fitchburg, WI, USA), and the number of apoptotic bodies in 10 randomly selected high-power fields was counted in a blinded manner.

### 2.9. Statistical Analysis

The data are presented as the mean ± standard error of the mean. The statistical significance of differences between treatment groups was assessed using the Mann–Whitney *U* test or analysis of variance with Bonferroni post hoc test. The data were analyzed using SPSS version 23.0 (IBM, Armonk, NY, USA). Statistical significance was defined as a two-sided *P* value <0.05.

## 3. Results and Discussion

### 3.1. Hederagenin Induces Apoptosis in Cisplatin-Sensitive and Cisplatin-Resistant HNC Cells

**Figure 1:**
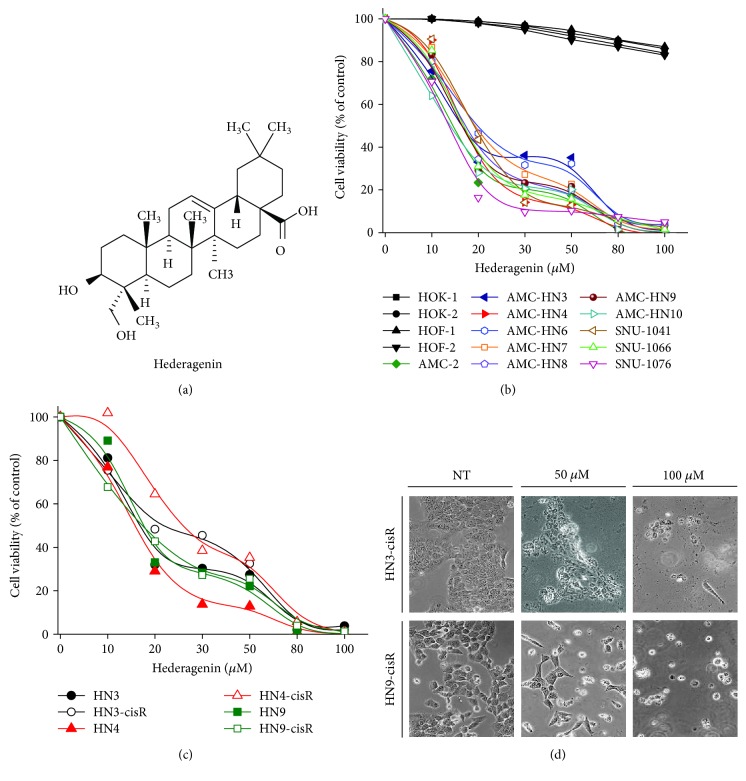
Hederagenin decreases the viability of HNC cells. (a) The structure of hederagenin. (b, c) Cell viability was assessed in cisplatin-sensitive and cisplatin-resistant HNC cell lines exposed to various concentrations of hederagenin for 72 h. (d) Images of hederagenin-treated and hederagenin-nontreated (NT) cisplatin-resistant HNC cells.

The molecular weight of hederagenin is 472.7 g/mol (Figure 1(a)). Hederagenin decreased the viability of cisplatin-sensitive and cisplatin-resistant cancer cells in a dose-dependent manner (Figures 1(b) and 1(c)). The viability decreased by up to 50% in cells treated with 20 *μ*M hederagenin and by up to 90% in cells treated with 80 *μ*M hederagenin for 72 h. Cisplatin-resistant HNC cells were less sensitive to hederagenin treatment compared with cisplatin-sensitive HNC cells. However, the cell viability was nearly abolished in all the cisplatin-sensitive and cisplatin-resistant HNC cells treated with hederagenin concentrations > 80 *μ*M. Representative images of hederagenin-treated cells are presented in Figure 1(d).

**Figure 2:**
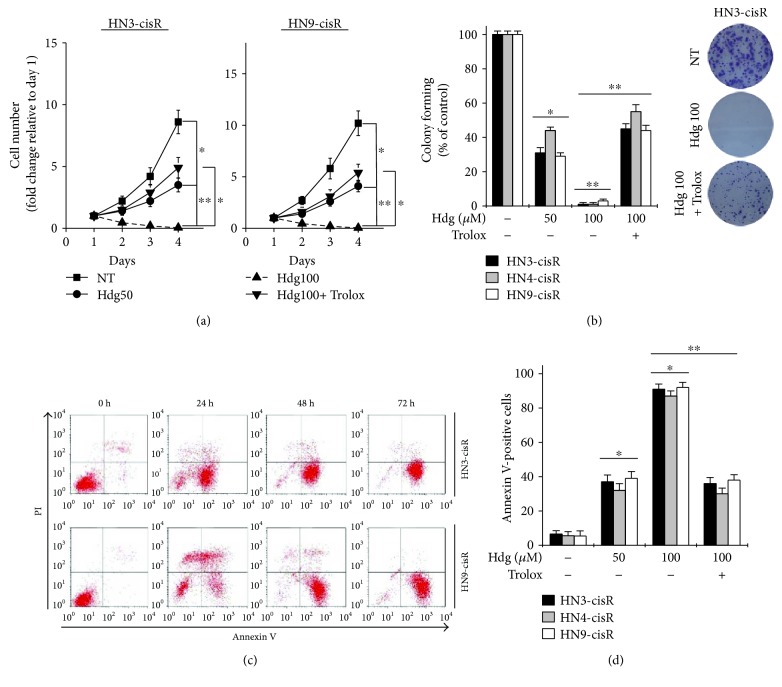
Hederagenin induces apoptotic cell death in cisplatin-resistant HNC cells. (a, b) Changes in cell number and colony-forming ability in cisplatin-resistant HNC cells exposed to hederagenin (Hdg) with or without pretreatment with the antioxidant trolox (0.5 mM). NT: control cells not treated with hederagenin. (c) FACS analyses of Annexin V and propidium iodide staining of cisplatin-resistant HNC cells treated with 100 *μ*M hederagenin. (d) Apoptosis in cells treated with various concentrations of hederagenin for 72 h was assessed by quantifying the proportion of Annexin V-positive cells. The error bars represent the standard error from three independent experiments. ^∗^*P* < 0.05, ^∗∗^*P* < 0.01 relative to the control or between groups.

Hederagenin inhibited the growth of cisplatin-resistant HNC cells in a treatment time- and dose-dependent manners (Figure 2(a)), and pretreatment with the antioxidant trolox (0.5 mM) inhibited this effect. In addition, hederagenin significantly suppressed colony formation in cisplatin-resistant HNC cells, and this effect was also significantly inhibited by trolox pretreatment (*P* < 0.05) (Figure 2(b)). Hederagenin induced apoptotic cell death in all cisplatin-resistant HNC cell lines evaluated. This effect was observed as early as 24 h after treatment, and it increased in a time-dependent treatment manner (Figures 2(c) and 2(d)).

Previous studies demonstrated that hederagenin induced cell death in human cancer cells by activating intrinsic apoptotic pathways [16–18]. A hederagenin saponin, macranthoside B, extracted from the Chinese plant *Lonicera macranthoides*, exerted cytotoxic effects in cancer cells. It also exhibited anti-inflammatory activity and provided protection against liver damage [18, 20]. The strong antitumor effect of hederagenin saponins is mediated by the activation of mitochondria-mediated apoptosis in hepatocellular carcinoma cells [18]. Hederagenin also strongly upregulates the apoptotic protein Bax and downregulates the antiapoptotic protein Bcl-2, thereby increasing the Bax/Bcl-2 ratio in cancer cells [18]. Hederagenin isolated from ivy leaves (*Hedera helix L*.) also induced apoptosis via the mitochondrial pathway in colon cancer cells [16]. Hederagenin activates the apoptosis executioner caspases, caspase-3, caspase-6, and caspase-9, thereby promoting cytochrome c release, but it does not activate caspase-8, a protein associated with the extrinsic apoptosis pathway [16, 21]. Hederagenin extracted from the leaves of the Chinese sweet tea *Cyclocarya paliurus* also selectively exerted cytotoxic effects in breast and lung cancer cells [17]. Hederagenin induces mitochondria-driven apoptosis and anti-inflammatory effects by suppressing the NF-*κ*B pathway, similar to the action of NF-*κ*B inhibitors [17]. Consistent with the previous findings, the present study demonstrated that the prooxidant effect of hederagenin selectively induced apoptosis in cisplatin-resistant cancer cells while sparing normal cells.

### 3.2. Hederagenin Induces Cellular GSH Depletion and ROS Accumulation in HNC Cells

**Figure 3:**
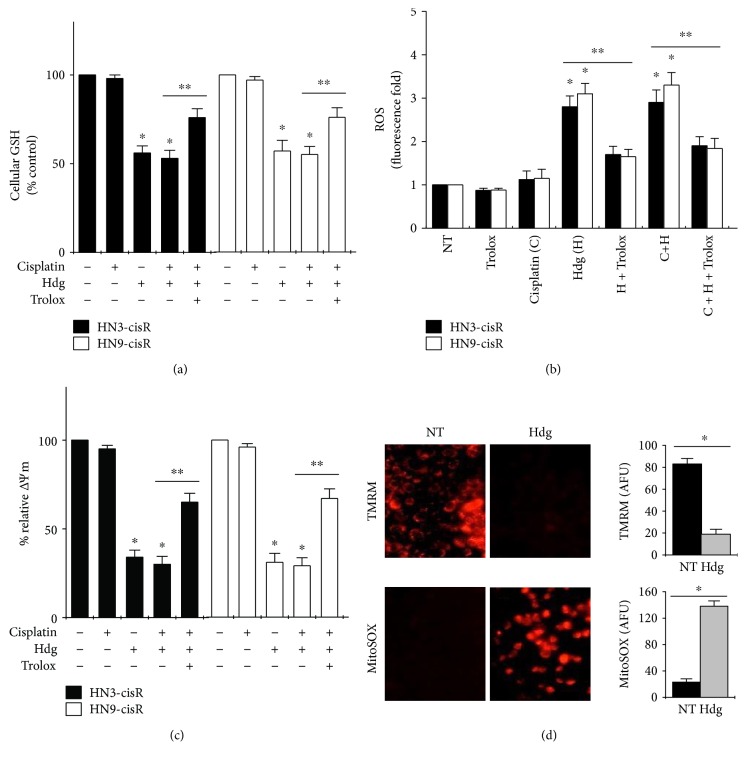
Hederagenin induces GSH depletion and ROS accumulation in cisplatin-resistant HNC cells. (a, b) Cellular GSH and ROS levels in cells treated with various combinations of 10 *μ*M cisplatin, 80 *μ*M hederagenin, and 0.5 mM trolox for 24 h. (c, d) Changes in the mitochondrial membrane potential (Δ*Ψ*m) in cisplatin-resistant HNC cells treated with various combinations of 10 *μ*M cisplatin, 80 *μ*M hederagenin, and 0.5 mM trolox for 24 h. Δ*Ψ*m was measured using flow cytometry analysis of cells stained with TMRE. The MFI of each treatment group was normalized to the control group. The error bars represent the standard error from three independent experiments. ^∗^*P* < 0.05 relative to control, ^∗∗^*P* < 0.05 between groups.

Cellular GSH levels significantly decreased and cellular ROS levels significantly increased in hederagenin-treated cells (*P* < 0.05) (Figures 3(a) and 3(b)), and these effects were significantly inhibited by pretreatment with 0.5 mM trolox (*P* < 0.05). Cisplatin treatment alone did not affect the cellular levels of GSH and ROS. In addition, hederagenin induced changes in Δ*Ψ*m in cisplatin-resistant HNC cells, as demonstrated by a decrease in TMRM staining and an increase in mitoSOX staining (Figures 3(c) and 3(d)). This effect was reversed by trolox pretreatment, and cisplatin alone did not affect Δ*Ψ*m.

Our study focused on the effect of hederagenin on the modulation of cellular oxidation. Hederagenin enhanced ROS production in HNC cells by promoting GSH depletion, and this effect was reversed by the antioxidant trolox. Consistent with the previous findings, the present study demonstrated that hederagenin induces apoptosis in cancer cells by downregulating Δ*Ψ*m. We also demonstrated that hederagenin activates regulator upstream of the intrinsic apoptotic pathway in HNC cells.

### 3.3. Hederagenin Inhibits the Nrf2-ARE Pathway

**Figure 4:**
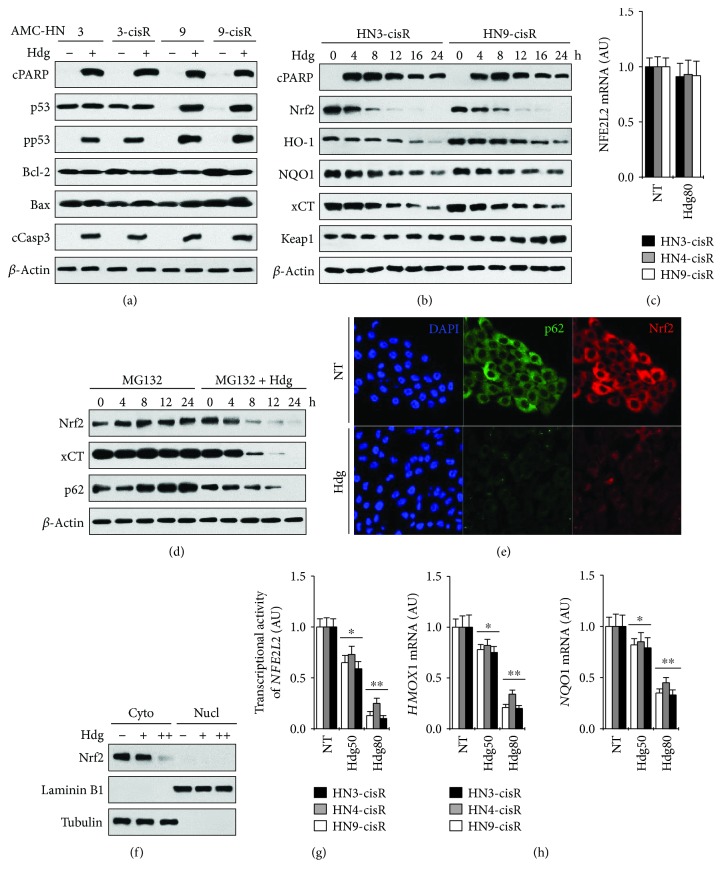
Hederagenin induces apoptosis by inhibiting the Nrf2-ARE pathway in HNC cells. (a, b) Western blot analysis of cleaved PARP (cPARP), p53, phospho-p53-Ser15 (pp53), Bcl-2, Bax, cleaved caspase-3 (cCasp3), Nrf2, HO-1, NQO-1, xCT, and Keap1 in HNC cells exposed to 80 *μ*M hederagenin for 24 h (a) or the indicated period of time (b). *β*-Actin was used as a loading control. (c) Quantitative real-time PCR analysis of *Nrf2* expression in cisplatin-resistant HNC cells exposed to 80 *μ*M hederagenin for 24 h. (d) Western blot analysis of Nrf2, xCT, and p62 in HN3-cisR cells treated with 80 *μ*M hederagenin and/or the proteasome inhibitor MG132 (5 *μ*M). (e) Immunofluorescence staining of p62 (green) and Nrf2 (red) in nontreated HN3-cisR cells and HN3-cisR cells treated with 80 *μ*M hederagenin for 24 h. DAPI was used as a nuclear counterstain. (f) Nrf2 levels in cytoplasmic and nuclear extracts of HN3-cisR cells exposed to 0, 50, or 80 *μ*M hederagenin for 24 h. (g) Nrf2 transcriptional activity in cisplatin-resistant HNC cells treated with 0 (NT), 50, or 80 *μ*M hederagenin for 24 h. (h) Changes in *HO-1* and *NQO1* mRNA levels in cisplatin-resistant HNC cells treated with 0 (NT), 50, or 80 *μ*M hederagenin for 24 h. The error bars represent the standard error from three replicate experiments ^∗^*P* < 0.05, ^∗∗^*P* < 0.01 relative to the NT control.

Hederagenin enhanced the levels of proapoptotic proteins (cleaved PARP, cleaved caspase-3, and BAX), whereas it suppressed the levels of the antiapoptotic protein Bcl-2. Hederagenin enhanced p53 levels in HN9 and HN9-cisR cells, which express wild-type p53 (Figure 4(a)), whereas it did not significantly affect p53 levels in HN3 or HN3-cisR cells, which express mutant p53 (R282W). However, phospho-p53 levels increased in hederagenin-treated HN3 and HN3-cisR cells. In addition, levels of the Nrf2-ARE antioxidant pathway components Nrf2, HO-1, NQO1, and xCT decreased in cisplatin-resistant HNC cells treated with hederagenin (Figure 4(b)), whereas *Nrf2* mRNA expression was not significantly affected (*P* > 0.1) (Figure 4(c)). The level of Keap1 increased in the HNC cells by hederagenin treatment along with the decreased level of p62. Regardless of treatment in combination with the proteasome inhibitor MG132, hederagenin decreased the levels of Nrf2, xCT, and p62 in a time-dependent manner (Figure 4(d)). Hederagenin-induced changes in Nrf2 and p62 were confirmed by the results of immunofluorescence staining assays (Figure 4(e)). Immunoblotting analysis of the cytoplasmic and nuclear extracts of HNC cells demonstrated that changes in Nrf2 levels in cisplatin-resistant HNC cells treated with hederagenin primarily occurred in the cytoplasm (Figure 4(f)). Hederagenin also inhibited the Nrf2 transcriptional activity and *HO-1* and *NQO1* mRNA levels in HNC cells (Figures 4(g) and 4(h)).

**Figure 5:**
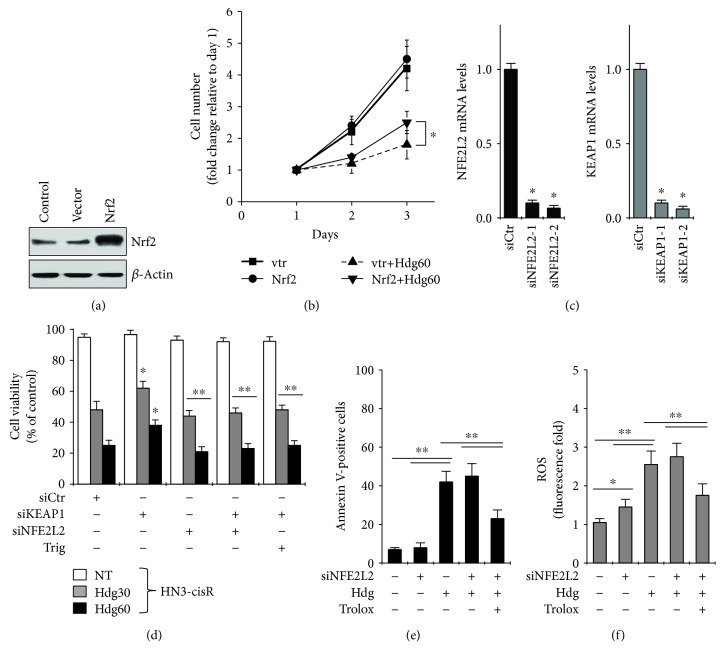
Hederagenin induces cell death in HNC cells by inhibiting Nrf2. (a, b) Effects of *Nrf2* overexpression on hederagenin-induced changes in cell growth. (c, d) Cell viability in HN3-cisR cells transfected with siControl (siCtr), si*NFE2L2*, or si*KEAP1* and exposed to hederagenin in the presence or absence of trigonelline (Trig, 100 *μ*M). ^∗^*P* < 0.05 relative to the siCtr; ^∗∗^*P* < 0.05 relative to the si*KEAP1* group with 30 *μ*M or 60 *μ*M hederagenin treatment. (e, f) FACS analysis and ROS levels in HN3-cisR cells transfected with siCtr or si*NFE2L2* and treated with 60 *μ*M hederagenin, 0.5 mM trolox, or both. Error bars represent the standard error from three replicate experiments. ^∗^*P* < 0.05, ^∗∗^*P* < 0.01 relative to the control or between groups.

*Nrf2* overexpression suppressed the inhibitory effects of hederagenin on cell growth, but it did not accelerate the growth of HNC cells in the absence of hederagenin (Figures 5(a) and 5(b)). *Nrf2* overexpression resulting from siRNA-mediated *Keap1* knockdown also suppressed the inhibitory effects of hederagenin on HNC cell growth (Figures 5(c) and 5(d)). However, neither siRNA-mediated *Nrf2* knockdown nor trigonelline-mediated pharmacological inhibition of Nrf2 enhanced the effects of hederagenin on cell viability, apoptosis, or cellular ROS accumulation (Figures 5(d), 5(e), and 5(f)). The effects of hederagenin on apoptosis and ROS accumulation were significantly inhibited in cells pretreated with trolox.

The current study demonstrated that hederagenin selectively induces HNC cell death by enhancing ROS production and promoting the depletion of GSH via inhibition of the Nrf2-ARE pathway. Nrf2 plays a central role in the cellular response to oxidative damage [22]. It regulates the expression of target genes associated with the cellular antioxidant systems that promote GSH production [14]. Nrf2 is constantly degraded by the proteosomal activity of Keap1; therefore, Nrf2 activity is upregulated by Keap1 inhibition [22, 23]. A growing body of evidence indicates that the Keap1-Nrf2 system plays an important role in carcinogenesis and chemotherapy resistance [24–27]. We previously demonstrated that Nrf2 plays a role in cisplatin resistance in HNC [28]. The present study demonstrated that cytoplasmic levels of Nrf2 increased in cisplatin-resistant HNC cells, consistent with its proposed association with tumor aggressiveness [29], and this effect was strongly inhibited by hederagenin treatment. In addition, we demonstrated that hederagenin upregulated wild-type p53 and phospho-p53 in HNC cells, an effect that partially suppressed Nrf2-dependent transcription of antioxidant response genes and activated proapoptosis proteins [30]. The effects of hederagenin on Nrf2 were observed in all of the tumor cells evaluated, regardless of the *P53* mutation status. As *P53* mutations can contribute to treatment resistance, this observation might partially account for the ability of hederagenin to effectively target cisplatin-resistant HNC cells.

Both siRNA-mediated *Nrf2* knockdown and pharmacological inhibition of Nrf2 markedly suppress GSH levels and enhance the cytotoxic effects of chemotherapeutic agents [26, 27]. Cancer cells are characterized by high levels of oxidative stress due to elevated ROS levels, and this defect alters various cellular metabolic pathways and activates antioxidant defense mechanisms [12, 13]. Therefore, oxidative stress might represent a critical weakness that can be targeted by selective therapeutic approaches to various types of cancer [14]. Combinations of antioxidant inhibitors and chemotherapeutic agents promote oxidative stress in cancer cells, thereby selectively promoting cell death in cancer cells [31, 32]. Therefore, elevated ROS levels are increasingly accepted as a valuable therapeutic target in anticancer drug discovery [14, 15]. We previously demonstrated that wogonin, a natural active flavonoid, inhibited Nrf2 upregulation in cisplatin-resistant HNC cells [28], similar to what has been observed with other anticancer drugs [26, 33]. In the present study, hederagenin inhibited the Nrf2-ARE pathway in cisplatin-resistant HNC cells, thereby inducing cytotoxic effects. Collectively, our results suggest that the Nrf2 pathway is a potential target of future therapies for chemoresistant HNC.

### 3.4. Hederagenin Inhibits the Growth of Cisplatin-Resistant HNC In Vivo

**Figure 6:**
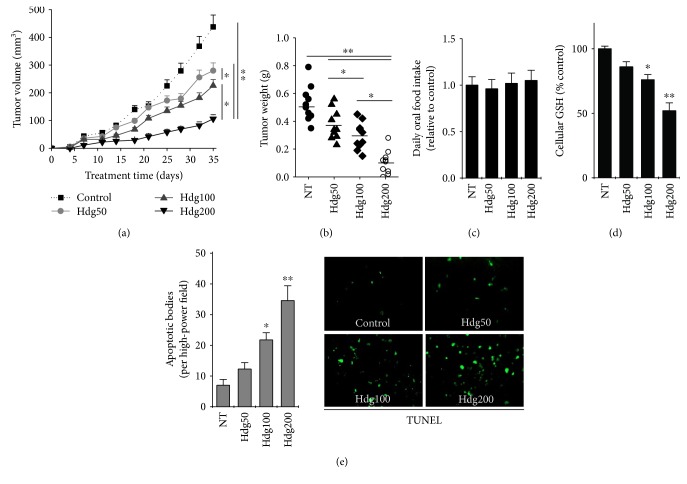
Hederagenin inhibits the growth of cisplatin-resistant HNC cell tumor xenografts. (a, b) Growth and weight of HN9-cisR tumors in nude mice. The mice received intraperitoneal injections of the vehicle control (NT) or 50, 100, or 200 mg/kg hederagenin. (c) Comparison of daily food intake among different treatment groups. (d) Cellular GSH levels in tumors treated with vehicle or hederagenin. (e) TUNEL-positive apoptotic bodies in 10 randomly selected high-power fields were counted in a blinded manner. The error bars represent standard errors. ^∗^*P* < 0.05, ^∗∗^*P* < 0.01 relative to the control or other treatment groups.

In mouse xenograft models injected with HN9-cisR cells, hederagenin significantly suppressed tumor growth *in vivo* in a dose-dependent manner (Figures 6(a) and 6(b)). Hederagenin did not significantly affect daily food intake or body weight compared with the vehicle control (Figure 6(c)). Hederagenin significantly suppressed GSH levels in tumor cells compared with the vehicle control (*P* < 0.05) (Figure 6(d)). In addition, the number of TUNEL-positive apoptotic bodies in tumors significantly increased in the hederagenin-treated group compared with that in the control group (*P* < 0.05) (Figure 6(e)). At the histological level, there were no significant differences in vital organs between the treatment groups (data not shown).

Hederagenin has been reported to exert a cytoprotective effect on normal tissues [34, 35]. A recent study demonstrated that hederagenin might prevent alcoholic liver injury via its anti-inflammatory and antiapoptotic activities [34]. The expression of apoptotic proteins and proinflammatory cytokines is lower in normal cells than in cancer cells. Hederagenin demonstrated therapeutic effects in neurodegenerative diseases such as Parkinson's disease and Huntington's disease by inducing autophagy and promoting the degradation of disease-associated proteins [35]. In the present study, hederagenin selectively induced apoptosis in cancer cells while sparing normal cells. Interestingly, hederagenin treatment was not associated with weight loss or histological changes in major organ systems. As evidence demonstrating the anticancer activity of hederagenin has been accumulated, novel hederagenin derivatives are being developed for use as potential anticancer agents [19]. Compared with the parental molecule, hederagenin derivatives appear to have a more potent effect on apoptosis induction in human cancer cell lines [19]. Therefore, hederagenin is more likely to be used as a triterpene template for the discovery of new anticancer compounds.

## 4. Conclusion

**Figure 7:**
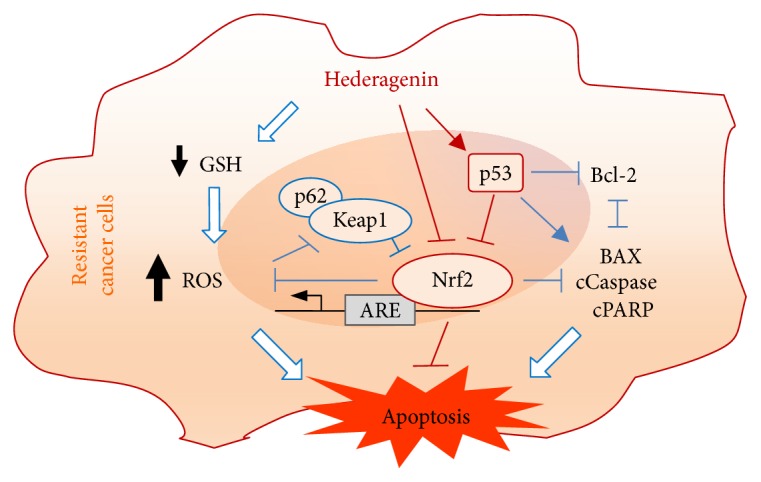
A model of the proposed mechanism of hederagenin in cisplatin-resistant HNC cells. Hederagenin selectively induces apoptosis in cancer cells by promoting ROS production and GSH depletion via inhibition of the Nrf2-ARE pathway.

The present study revealed a novel mechanism by which hederagenin enhances ROS levels in cisplatin-resistant cancer cells by inhibiting the Nrf2-ARE pathway, a central player in redox homeostasis (Figure 7). Our findings suggest that hederagenin effectively targets cisplatin-resistant HNC cells *in vitro* and *in vivo*. Consistent with its effects in other types of cancer, hederagenin markedly induces apoptosis in HNC cells by activating the mitochondria-driven intrinsic apoptotic pathway. We demonstrated that the apoptosis-inducing effects of hederagenin are mediated by the inhibition of the Nrf2-ARE antioxidant pathway. Additional preclinical and clinical investigations of this promising anticancer therapy in patients with other types of treatment-resistant cancer are warranted.

## Abbreviations

ARE: Antioxidant response element
DCF-DA: 2′,7′-Dichlorofluorescein diacetate
GSH: Glutathione
HNC: Head and neck cancer
HO-1: Heme oxygenase-1
Keap1: Kelch-like ECH-associated protein 1
MFI: Median fluorescence intensity
Δ*Ψ*m: Mitochondrial membrane potential
NQO1, NAD(P)H: Quinone oxidoreductase 1
Nrf2: Nuclear factor (erythroid-derived 2)-like 2
PARP: Poly(ADP-ribose) polymerase
PI: Propidium iodide
ROS: Reactive oxygen species
RT-qPCR: Reverse transcription-quantitative polymerase chain reaction
siRNA: Short interfering RNA
TMRE: Tetramethylrhodamine ethyl ester
TUNEL: Terminal deoxynucleotidyl transferase dUTP nick end labeling
xCT: Cystine/glutamate antiporter system xc^−^.
